# Estimating crop coefficients and actual evapotranspiration in citrus orchards with sporadic cover weeds based on ground and remote sensing data

**DOI:** 10.1007/s00271-022-00829-4

**Published:** 2022-10-15

**Authors:** Matteo Ippolito, Dario De Caro, Giuseppe Ciraolo, Mario Minacapilli, Giuseppe Provenzano

**Affiliations:** 1 Department of Agriculture, Food and Forest Sciences, University of Palermo, Viale delle Scienze Ed.4, 90128 Palermo, Italy; 2 Engineering Department, University of Palermo, Viale delle Scienze Ed. 8., 90128 Palermo, Italy

## Abstract

Accurate estimations of actual crop evapotranspiration are of utmost importance to evaluate crop water requirements and to optimize water use efficiency. At this aim, coupling simple agro-hydrological models, such as the well-known FAO-56 model, with remote observations of the land surface could represent an easy-to-use tool to identify biophysical parameters of vegetation, such as the crop coefficient K_c_ under the actual field conditions and to estimate actual crop evapotranspiration. This paper intends, therefore, to propose an operational procedure to evaluate the spatio-temporal variability of K_c_ in a citrus orchard characterized by the sporadic presence of ground weeds, based on micro-meteorological measurements collected on-ground and vegetation indices (VIs) retrieved by the Sentinel-2 sensors. A non-linear K_c_(VIs) relationship was identified after assuming that the sum of two VIs, such as the normalized difference vegetation index, NDVI, and the normalized difference water index, NDWI, is suitable to represent the spatio-temporal dynamics of the investigated environment, characterized by sparse vegetation and the sporadic presence of spontaneous but transpiring soil weeds, typical of winter seasons and/or periods following events wetting the soil surface. The K_c_ values obtained in each cell of the Sentinel-2 grid (10 m) were then used as input of the spatially distributed FAO-56 model to estimate the variability of actual evapotranspiration (ET_a_) and the other terms of water balance. The performance of the proposed procedure was finally evaluated by comparing the estimated average soil water content and actual crop evapotranspiration with the corresponding ones measured on-ground. The application of the FAO-56 model indicated that the estimated ET_a_ were characterized by root-mean-square-error, RMSE, and mean bias-error, MBE, of 0.48 and -0.13 mm d^−1^ respectively, while the estimated soil water contents, SWC, were characterized by RMSE equal to 0.01 cm^3^ cm^−3^ and the absence of bias, then confirming that the suggested procedure can produce highly accurate results in terms of dynamics of soil water content and actual crop evapotranspiration under the investigated field conditions.

## Introduction

Monitoring crop evapotranspiration (ET_c_) is crucial to evaluating actual crop water requirements. A widely accepted practical approach to estimating ET_c_ is the FAO-56 (Allen et al. 1998), in which ET_c_ is calculated as the product of crop reference evapotranspiration (ETo) and a single or dual crop coefficient (K_c_ or K_cb_ + K_e_). ETo represents the evapotranspiration of a reference hypothetical crop (i.e., alfalfa), actively growing, adequately watered and characterized by defined values of height, surface resistance, and albedo, which is, therefore, associated with the meteorological evaporative demand. The standard method to estimate ETo is the FAO-56 Penman–Monteith equation (Allen et al. 1998).

Due to its simplicity, the FAO-56 approach has been widely applied to various crops to estimate water requirements and to compute all the terms of soil water balance (SWB) in the root zone, based on a simplified reservoir scheme. The dynamic of water content in the soil root layer includes a corrective coefficient K_s_ (0–1) expressive of actual crop water stress conditions. Moreover, using the dual crop coefficient method, the FAO-56 approach accounts separately for plant transpiration (T) and soil evaporation (E), represented by a basal crop coefficient (K_cb_) and a soil evaporation coefficient (K_e_), respectively (Allen et al. 1998). Actual crop evapotranspiration, ET_a_, can be, therefore, estimated as the product of ETo and the term (K_cb_K_s_ + K_e_).

Although detailed equations and tabulated values for the crop coefficients have been suggested in the FAO-56 manual, it has been strongly recommended to check their suitability based on in situ measurements and local adaptations. As a result, in the last decade, the scientific community has revised the crop coefficients from the original values, also considering the specific crop varieties or climatic conditions, and adding K_c_ values for crops not originally considered (Pereira et al. 2015; Rallo et al. 2021). However, these coefficients are generally referred to specific conditions, such as the presence or the absence of active ground cover or weeds, and are assumed valid during the entire irrigation season, without including the possibility of time-variable conditions.

Despite the remote sensing (RS) technique has not been considered in the FAO-56 method, in the past two decades, several studies have been proposed to take advantage of multispectral remotely sensed images to estimate the spatio-temporal variability of K_c_ or K_cb_ to support irrigation management (e.g., Bausch and Neale 1987; Bausch 1995; Campos et al. 2010, 2017; Choudhury et al. 1994; Er-Raki et al. 2007; González-Dugo et al. 2013; Hunsaker et al. 2003; Mateos et al. 2013; Neale et al. 1989; Padilla et al. 2011; Pôças et al. 2015). RS technologies represent a useful tool to quantify various vegetation parameters such as albedo, surface temperature, crop coefficients, and leaf area index, with the advantage to capture their spatial and temporal variability at different scales. Regarding the crop coefficient K_c_, two different RS techniques have been proposed. The first one is analytical, and based on the direct application of the Penman–Monteith equation in which input data for crop characterization (Leaf Area Index, LAI, height and albedo) are estimated using multispectral images operating in the visible and near-infrared (VIS–NIR) domain (D’Urso 2001; Minacapilli et al. 2008); the second technique is based on a vegetation indices (VI-K_c_) approach, based on the assumption of a direct relationship between K_c_ and various vegetation spectral indices (i.e., NDVI, SAVI, EVI, etc.) derived from reflectance in the VIS–NIR, domain (Gontia and Tiwari 2010; Er-Raki et al. 2013; Kamble et al. 2013; Alam et al. 2018). An overview of the RS data and missions mostly used to implement the K_c_–VI approaches, running from satellite missions with long imagery archives until new satellite programs and constellations, has been recently proposed by Pôças et al. (2020). The main advantage of the VI–K_c_ approach is that the vegetation indices operating in the VIS–NIR domain are readily available; on the other hand, the VI–K_c_ approach is based on crop-specific regressions, whose assessment requires calibration and validation procedures.

Pôças et al. (2020) reported an exhaustive list of different K_c_–VI relationships valid for a variety of crops and advocated their advantages and limitations based on a SWOT analysis. If on the one hand, the authors showed the performance of different K_c_–VI relationships based on the comparisons between estimated and measured ET_c_ reported in various studies, which were characterized by root mean square errors (RMSE) ranging between 0.40 and 0.72 mm d^−1^ or root mean square differences (RMSD) ranging between 0.50 and 1.00 mm d^−1^. On the other hand, they stated that the examined approaches have a local validity and also that complementary methodology may be required to adjust for the actual field conditions. Moreover, most of these studies were related to herbaceous crops that uniformly cover the soil surface, while only a few investigations have been carried out on sparse vegetation; for these cases, experimental research is still necessary to verify the reliability of the K_c_ values suggested in the literature, especially for sparse tree crops such as citrus orchards, characterized by the presence of active ground weeds that can temporarily spread over the soil surface (Saitta et al. 2020). Citrus orchards are extensively diffused in the Mediterranean regions and have an important role in the economy of these areas. Usually, weeds will germinate from the seed immediately after wetting the soil surface due to the occurrence of rainfall or surface irrigation events. The presence of weeds, even if temporary, can contribute to the reduction of water and nutrients in the root zone, limiting crop availability and penalizing crop yield (Singh et al. 2022). The knowledge of the contribution of weed transpiration to the spatio-temporal variability of water depletion is crucial to improving the performance of water balance models and optimizing irrigation water management (Zimdahl 2018).

Sicily island (Southern Italy), characterized by a semi-arid climate, has excellent potential for citrus production. The region is characterized by scarce precipitation in the period from April to September, during which citrus orchards need to be irrigated; the assessment of the correct crop water requirement is, therefore, of utmost importance.

The main objectives of this work were (i) to estimate reliable K_c_ values for a typical Sicilian citrus orchard characterized by temporary ground weeds contributing to soil water depletion; (ii) to develop a functional relationship between the crop coefficient and remote sensed vegetation indices to assess the spatial and temporal variability of the crop coefficient during the different crop growth stages; and iii) to assess the suitability of the proposed approach, based on the performance of the FAO-56 model to estimate soil water contents and actual crop evapotranspiration.

The estimated values of actual evapotranspiration, ET_a_, and soil water content, SWC, aggregated at plot levels, were finally compared with the corresponding measured on-site. The comparative analysis indicated that the estimated ET_a_ values were characterized by average root-mean-square-error, RMSE, and mean bias-error, MBE, are equal to 0.48 and -0.13 mm d^−1^ respectively, while the estimated SWC resulted in an RMSE equal to 0.01 cm^3^ cm^−3^ and the absence of bias.

## Materials and methods

### Description of the study area and experimental layout

The experiment was carried out in a citrus orchard of about 0.4 ha located in Palermo, Sicily (38°4′ 53.4’’ N, 13° 25′ 8.2’’ E) in the period from 2018 to 2020.

**Fig. 1:**
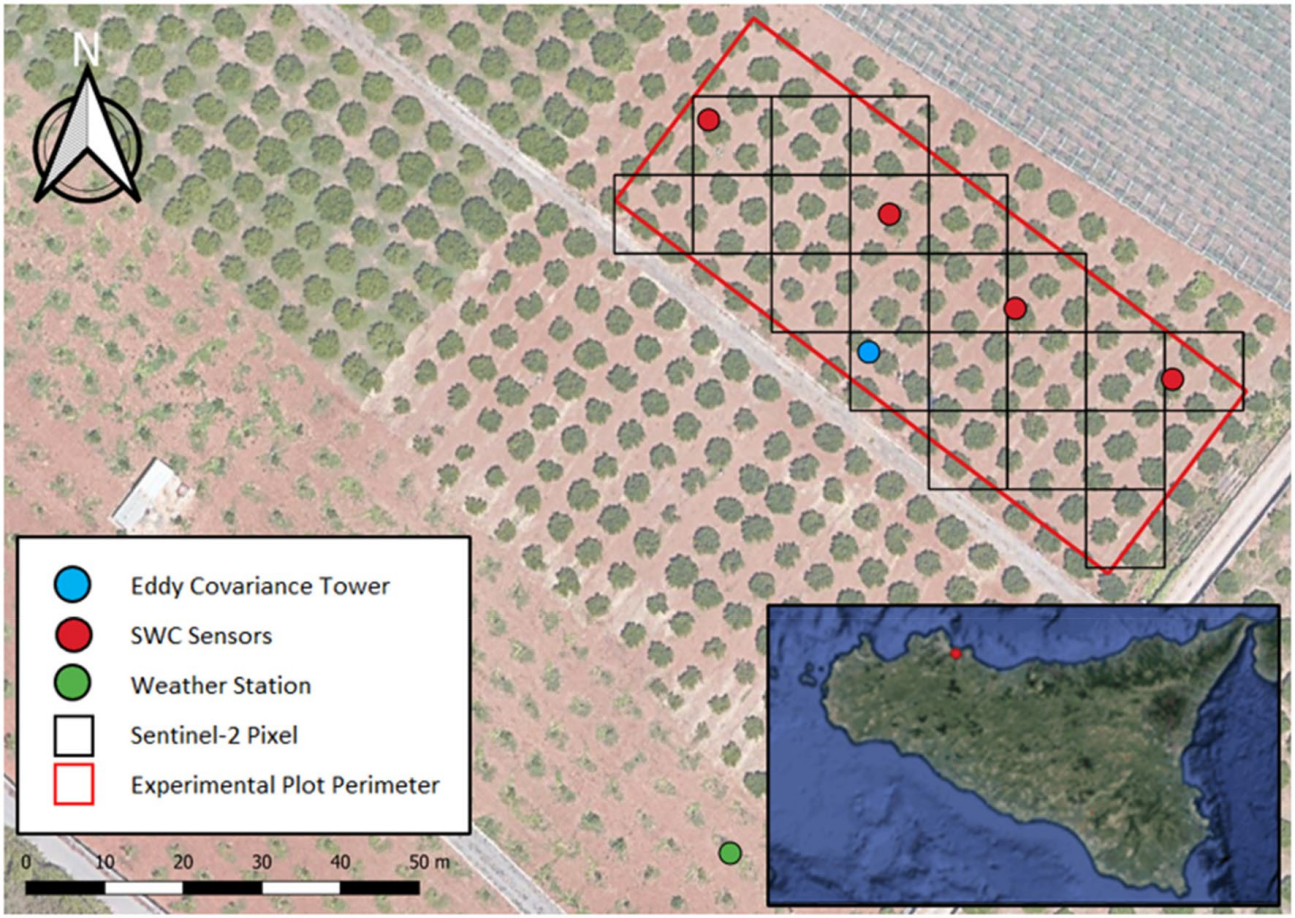
Experimental field with location of weather station (WS), flux tower (EC), and soil water content sensors; the spatial resolution of Sentinel-2 (L2A/L2B) multispectral images is also shown

The climate of the investigated area is the typical Mediterranean, with annual rainfall ranging between 600 and 800 mm, most of which is concentrated in fall and winter, and cumulated crop reference evapotranspiration generally higher than 1000 mm. The average daily air temperature ranges from about 4 °C in winter to a maximum of around 35 °C in summer. Figure 1 shows the location of the orchard and a map of the experimental field with the position of the installed equipment and facilities. The citrus orchard (*Citrus reticulata Blanco,* cv. Mandarino Tardivo di Ciaculli) is characterized by planting spacing of 5.0 × 5.0 m (density of 400 trees ha^-1^, fraction cover of 48%), with plant rows roughly oriented from North-East to South-West. The trees are characterized by an average height of about 2.5 m and a maximum rooting depth of 0.5 m, with the highest root density around 0.3 m depth. The dominant textural class of the topsoil is sandy-clay-loam with average clay, silt and sand content of 22.2%, 18.0% and 59.8%, respectively. Soil water contents at field capacity (*SWC*_*fc*_) and permanent wilting point (*SWC*_*wp*_) are equal to 0.28 cm^3^cm^−3^ and 0.15 cm^3^cm^−3^, respectively. During the irrigation seasons, generally after the rainfall events, the field was characterized by the presence of temporary ground weeds (mainly Cynodon Dactylon, and Boerhavia Coccinea).

Agrometeorological data were collected by a WatchDog 2000 series weather station (Spectrum Technologies, Inc., Aurora, IL, USA) installed nearby the experimental field (Fig. 1), including the sensors to measure, with a time-step of 30 min, air temperature, global solar radiation, relative air humidity, wind speed and direction at 2 m height, and rainfall.

Irrigation season in the area ordinarily starts at the end of May and finishes around the end of September, during periods with scarce or absent precipitations. The field is irrigated with a subsurface drip system with two pipes per plant row, one on each side of the tree, at 1.1 m from the trunks. The lateral pipes contain co-extruded emitters discharging 2.3 l h^-1^ at a pressure of 100 kPa with a spacing of 1.0 m (i.e., 10 emitters/tree). The adopted irrigation strategy, accounting for the actual climate conditions, was designed to define moderate crop water stress only during phase II of vegetative growth (initial fruit enlargement phase), generally occurring between July 1 and August 15, during which the lower threshold of Midday Stem Water Potential (MSWP) ranged between − 1.4 and − 2.0 MPa. During the other phases of crop growth, the values of MSWP ranged between − 0.4 and − 1.4 MPa. A total of three watering of about 56 m^3^ ha^-1^ were generally scheduled per week, except for the period of water restrictions, in which only two irrigation events were scheduled per week.

The temporal dynamic of soil water content (SWC) was monitored with four 0.6 m long "drill & drop" probes (Sentek Pty Ltd, Stepney, Australia), installed at a distance of 0.8 m from the tree trunks and 30 cm far from an emitter (Fig. 1). These sensors, based on the frequency-domain reflectometry (FDR) technique allowed monitoring of the soil water content and temperature at each 0.1 m depth, up to 0.6 m, with a time-step of about 30 min. The average SWC between the soil surface and 0.5 m depth was assumed representative of the entire root domain.

During each irrigation season, weekly measurements of Midday Stem Water Potential (MSWP, MPa) were carried out in the same four trees in which the SWC sensors were installed, with a pressure chamber (Plant Moisture Vessel SKPM 1400 series, Skye Instruments Ltd, Llandrindod Wells, Powys, UK), following the protocol described in Turner (1981). These data (not reported) allowed the identification of the critical threshold of SWC equal to about 0.21 cm^3^ cm^−3^, below which initiates the moisture stress, with reduction of crop transpiration.

In 2019, an Eddy Covariance flux tower (EC) was also installed in the field to measure actual evapotranspiration, ET_a_ (Fig. 1). The tower was equipped with a 4-component net radiometer (CNR4, Campbell Scientific Inch., Logan, Utah) installed at 3.0 m height to measure low-frequency (30 min) net radiation, R_n_ [W m^−2^], a three-dimensional sonic anemometer (CSAT3-D, Campbell Scientific Inch., Logan, Utah) to measure high-frequency (20 Hz) wind speed 3D components, and an infrared open patch gas analyzer (Li-7500, Li-cor bioscience inch., Lincoln, Nebraska) to measure H_2_0 and CO_2_ concentrations, respectively (at 20 Hz). High and low-frequency data were collected in a CR3000 datalogger (Campbell Scientific Inch., Logan, Utah) equipped with a 2 GB memory card.

Sensible, H [W m^−2^] and latent, λET [W m^−2^] heat fluxes were evaluated as:

1 $$\,H = \rho c_{p} \sigma_{WT} ,$$

2 $$\lambda ET = \lambda \sigma_{WQ},$$

where ρ [g m^-^^3^] is the air density, c_p_ [J g^−1^ K^−1^] is the air-specific heat capacity at constant pressure and σ_WT_ [m K s^−1^] is the covariance between vertical wind speed and air temperature, λ [J g^−1^] is the latent heat of vaporization and σ_WQ_ [g m^−2^ s^−1^] is the covariance between vertical wind speed and the water vapor density. The row data acquired by the EC tower were processed using the software developed by Manca (2003), which allowed to determine the evapotranspiration fluxes with a time-step of 30 min, and then aggregated at a daily time-step to estimate daily ET_a_ [mm d^−1^].

Finally, an extended database of high-resolution multispectral images (MSI) retrieved from the Sentinel-2 twin satellites (L2A/L2B) was acquired to monitor, over the study area, the spatio-temporal variability of a set of VIS–NIR–SWIR vegetation indices. The product downloaded was the Multispectral Instruments (MSI) level 2A (ESA, https://scihub.copernicus.eu/) which provides images calibrated in reflectance at the bottom of the atmosphere (BoA), orthorectified and corrected for the atmospheric effects (Main-Knorn et al. 2017). The images have a spatial resolution of 10 m in the VIS–NIR regions and 20 m in the SWIR region, with a temporal resolution of approximately 5 days when considering both satellites (2A and 2B satellites). For the three years, a total of 193 scenes were selected under clear-sky conditions, downloaded and pre-processed using the R library named “sen2r” (Ranghetti et al. 2020),

### Estimating the crop coefficients from remote sensing data and ground measurements.

The onset of K_c_–VI approaches to estimate the crop coefficients relied on the evident similarities between the temporal patterns of K_c_ and VIs, such as the Normalized Difference Vegetation Index, NDVI, or the Soil Adjusted Vegetation Index, SAVI, found in pioneering studies (Bausch 1993; Bausch and Neale 1987; Heilman et al. 1982; Neale et al. 1989). Following these studies, a large set of relationships of VIs with either K_c_ or K_cb_ have been proposed.

As recently reported in Pôças et al. (2020), the VIs considered in the various linear or non-linear K_c_–VI relationships are only based on the Visible and Near-Infrared (VIS–NIR) reflectance; the most used VI is NDVI which can be calculated as (Rouse et al. 1974):

3 $$NDVI = \frac{{\rho_{nir} - \rho_{red} }}{{\rho_{nir} + \rho_{red} }},$$

where ρ_nir_ and ρ_red_ are the near-infrared and red reflectance. Similarly to the crop coefficient, NDVI is well correlated to the plant vigor, leaf area index and fraction cover.

The recent availability of high-resolution multispectral images from the ESA Sentinel-2 mission has allowed the easy recovery of other vegetation indices based on the shortwave region, SWIR, in addition to VIS–NIR. For the investigated case, the use of the SWIR region with the Normalized Difference Water Index (NDWI) has the advantage to be sensitive to the surface water content (Gao, 1996). The values of NDWI can be calculated as:

4 $$NDWI = \frac{{\rho_{nir} - \rho_{swir} }}{{\rho_{nir} + \rho_{swir} }},$$

where ρ_swir_ is the shortwave reflectance. Considering the different spatial resolutions associated with NIR and SWIR, to evaluate NDWI, the values of reflectance related to four pixels corresponding to the NIR domain were associated with a single pixel reflectance in the SWIR. The wavelengths, for Sentinel-2A and 2B satellites, are centered at 664.6 nm and 664.9 nm for the red band (B4), at 832.8 nm and 832.9 nm for NIR (B8) and 1613.7 nm and 1610.4 nm for SWIR (B11), respectively.

For the considered period (2018–2020), after retrieving the Sentinel-2 clear-sky images, a gap-filled database of daily NDVI and NDWI maps was generated using a linear interpolation technique (Pan et al. 2017) implemented in Matlab® R2019b and then exported in QGIS (release 3.4.3) environment.

A K_c_–VI non-linear relationship was initially identified by using an extended time series of multispectral images retrieved by the Sentinel-2 platform combined with a set of field micro-meteorological measurements. For the investigated orchard, the proposed K_c_–VI relationship allowed obtaining a *priori* a database of daily K_c_ maps characterized by high spatial resolution (10 m). The empirical relationship was obtained in the absence of crop water stress and, therefore, on days in which SWC in the orchard resulted higher than 0.21 cm^3^cm^−3^, which was identified as the critical threshold of soil water content (SWC^*^) below which crop water stress occurs in the orchard (Franco et al. 2022). The database of daily K_c_ maps was then used as input for the FAO-56 model to estimate the spatio-temporal variability of actual evapotranspiration, as well as the other terms of water balance in the root zone.

### FAO-56 model to estimate crop evapotranspiration

The FAO-56 model suggests the use of the Penman–Monteith (PM) equation for estimating daily crop reference evapotranspiration, ETo [mm d^−1^] (Allen et al. 1998):

5 $$ET_{0} = \frac{{0.408 \Delta \left( {R_{n} - G} \right) + \gamma \left( {\frac{900}{{T_{a} }} + 273} \right)\left( {U_{2} \left( {e_{s} - e_{a} } \right)} \right)}}{{\Delta + \gamma \left( {1 + 0.34U_{2} } \right)}} ,$$

where Δ [kPa °C^-1^] is the slope of saturation vapor pressure curve, *R*_*n*_ [MJ m^−2^ d^−1^] is the net radiation at the crop surface, *G* [MJ m^−2^ d^−1^] is the soil heat flux density, (*e*_*s*_–*e*_*a*_) [kPa] is the actual vapor pressure deficit, γ [kPa °C^−1^] is the psychrometric constant and *U*_*2*_ [m s^−1^] is the wind speed measured at 2 m height.

Based on the daily reference evapotranspiration values, the FAO-56 model estimates daily crop potential evapotranspiration, ET_c_, by multiplying ETo with a crop coefficient, K_c_, accounting for the differences between the biophysical characteristics of the reference crop (canopy properties, ground cover, aerodynamic resistance) and the considered crop. The FAO-56 model can be applied based on a single or a dual crop coefficient approach. In the former, soil evaporation and crop transpiration are merged into a single K_c_ value for each crop stage; in the latter, crop transpiration is estimated using a basal crop coefficient (K_cb_), whereas soil evaporation is based on the evaporation coefficient (K_e_). Thus, ET_c_ is, therefore, split into potential crop transpiration (T_c_ = K_cb_ ETo) and soil evaporation (E_s_ = K_e_ ETo).

The standard tabulated values of K_c_ and K_cb_ are generally used to estimate ET_c_ under potential and well-watered conditions. However, under actual field conditions, the crop is often subjected to water stress due to limited irrigation doses or inappropriate management practices. Thus, in the FAO-56 model a water stress coefficient, K_s_, ranging between 0 and 1, is introduced as a multiplicative factor to estimate the actual values of K_c_ or K_cb_. Consequently, actual crop evapotranspiration, ET_a_, is generally smaller than the corresponding potential and can be defined as:

6 $${\text{ET}}_{{\text{a}}} {\text{ = (K}}_{{\text{s}}} {\text{ K}}_{{\text{c}}} {\text{) ETo,}}$$

if the single crop coefficient approach is used, or

7 $${\text{ET}}_{{\text{a}}} {\text{ = (K}}_{{\text{s}}} {\text{ K}}_{{{\text{cb}}}} {\text{ + K}}_{{\text{e}}} {\text{) ETo,}}$$

if the dual crop coefficient approach is used.

The water stress factor K_s_ is generally estimated as a linear function of the root zone depletion D [mm]. In the absence of crop water stress (D ≤ RAW) K_s_ = 1, whereas under soil water deficit conditions (D > RAW) it can be evaluated as a linear function of the soil water depletion D [mm] (Allen et al. 1998):

8 $$K_{s} = \frac{TAW - D}{{TAW - RAW}},$$

where *TAW* and *RAW* [mm] are the total and readily available water in the root zone, respectively, with *RAW* = *p TAW*, being *p* the fraction of soil water depletion identifying the absence of crop water stress. According to Eq. 6, K_s_ < 1.0 when the root zone depletion exceeds RAW, i.e., the water depleted fraction is larger than *p*. Typical depletion coefficients, *p*, for various crops type are tabulated in the FAO-56 manual.

At the daily time-step, the soil water balance in the root zone, Z_r_ [m], can be written as:

9 $$D_{i} = D_{i - 1} - \left( {P_{i} - Ro_{i} } \right) - I_{i} + ETa_{i} + DP_{i} ,$$

where D_i_ and D_i−1_ [mm] are the root zone depletions at the end of the day i and i-1, P_i_ [mm] is the net precipitation, Ro_i_ [mm] is the surface runoff, ETa_i_ [mm] is the actual crop evapotranspiration, I_i_ [mm] is the irrigation depth and DP_i_ [mm] is the deep percolation of water moving out of the root zone.

Net precipitation was calculated by reducing the gross precipitation P [mm] of the canopy interception, P_in__t_ [mm], estimated as (Braden 1985):

10 $$P_{int} = a LAI \left( {1 - \frac{1}{{1 + \frac{b P}{{a LAI}}}}} \right) ,$$

where *a* is an empirical conversion coefficient, and *b* is the soil cover fraction, corresponding to about LAI/3. For ordinary crops, it is possible to assume *a* = 0.25.

The domain of the depletion ranges between 0, occurring when the soil is at the field capacity, to a maximum value corresponding to the total available water (TAW, mm), evaluated as:

11 $$TAW = 1000 \left( {SWC_{fc} - SWC_{wp} } \right) Z_{r} ,$$

where SWC_*fc*_ [cm^3^ cm^−3^] and SWC_*wp*_ [cm^3^ cm^−3^] are the soil water contents at field capacity and wilting point, respectively, and Z_*r*_ [m] is the rooting depth.

Model simulations to estimate daily values of soil water content and actual crop evapotranspiration and to assess the performance of the crop coefficients estimated with remote sensing acquisitions were carried out during three irrigation seasons, from DOY 137 to DOY 273, by considering, as initial soil water content (SWC_*0*_) the corresponding measured. Table 1 summarizes the values of the input variables assumed for the model simulations and the related data sources.

**Table 1:** Values for the variables used for FAO-56 model simulations

Variable	Units	2018	2019	2020	Data source
SWC_*fc*_	[cm^3^cm^−3^]	0.28	0.28	0.28	Measured
SWC_*wp*_	[cm^3^cm^−3^]	0.15	0.15	0.15	Measured
SWC_0_	[cm^3^cm^−3^]	0.20	0.23	0.24	Measured
SWC^*^	[cm^3^cm^−3^]	0.21	0.21	0.21	Fixed
*Z*_*r*_	[m]	0.50	0.50	0.50	Fixed

### Statistical analysis for model validation

The model performance was evaluated based on the following goodness-of-fit indicators used to assess the matching between measured and estimated soil water contents and actual evapotranspiration: Root Mean Square Error (RMSE, cm^3^ cm^−3^ or mm d^−1^), whose target value is zero when there are no differences between simulated and observed values; Mean Bias Error (MBE cm^3^ cm^−3^ or mm d^−1^), whose target value is zero; a positive value indicates that simulated values are overestimated, while a negative value indicates the model underestimation (Kennedy and Neville 1986); the regression coefficient (b, dimensionless), whose target value is one; represents the angular coefficient of the regression line between simulated and observed variables forced to the origin; the coefficient of determination (R^2^, dimensionless) whose target value is one, indicating that the variance of the observed values is explained by the model (Eisenhauer 2003). The percent bias (PBIAS, %), whose target value is zero; positive values are associated with the model underestimation, while negative values indicate the model overestimation; the Nash–Sutcliffe efficiency coefficient (NSE, dimensionless), whose target value is one; values between 0.0 and 1.0 indicate an acceptable model performance, whereas negative values indicate that the mean of observed values is a better predictor than the simulated values and, therefore, unacceptable performance (Nash and Sutcliffe 1970).

## Results

### In situ measurements

For the three years of observation (2018–2020), Fig. 2 shows the temporal dynamics of precipitation, P, the amount of irrigation, I, and the daily crop reference evapotranspiration, ETo, estimated with Eq. 5. The gray boxes identify the irrigation seasons (light gray), which include the periods of water deficit application (dark gray), usually applied from the beginning of July to mid-August. As it can be observed, even if the annual trends of daily ETo during the three years resulted quite similar, the values in 2018 were relatively lower than in the other two years. The maximum crop reference evapotranspiration generally occurred in July, during periods of limited or absent precipitation, while the minimum was registered between December and January. On the other hand, a certain variability during the three years can be noticed in the patterns of rainfall; the number of rainy days, with rainfall value equal to or higher than 2.5 mm d^−1^, resulted equal to 65 in 2018 and only 44 in 2020, when a prolonged drought period occurred at the beginning of the year, followed by an extreme event of 97.3 mm registered on March 25.

**Fig. 2:**
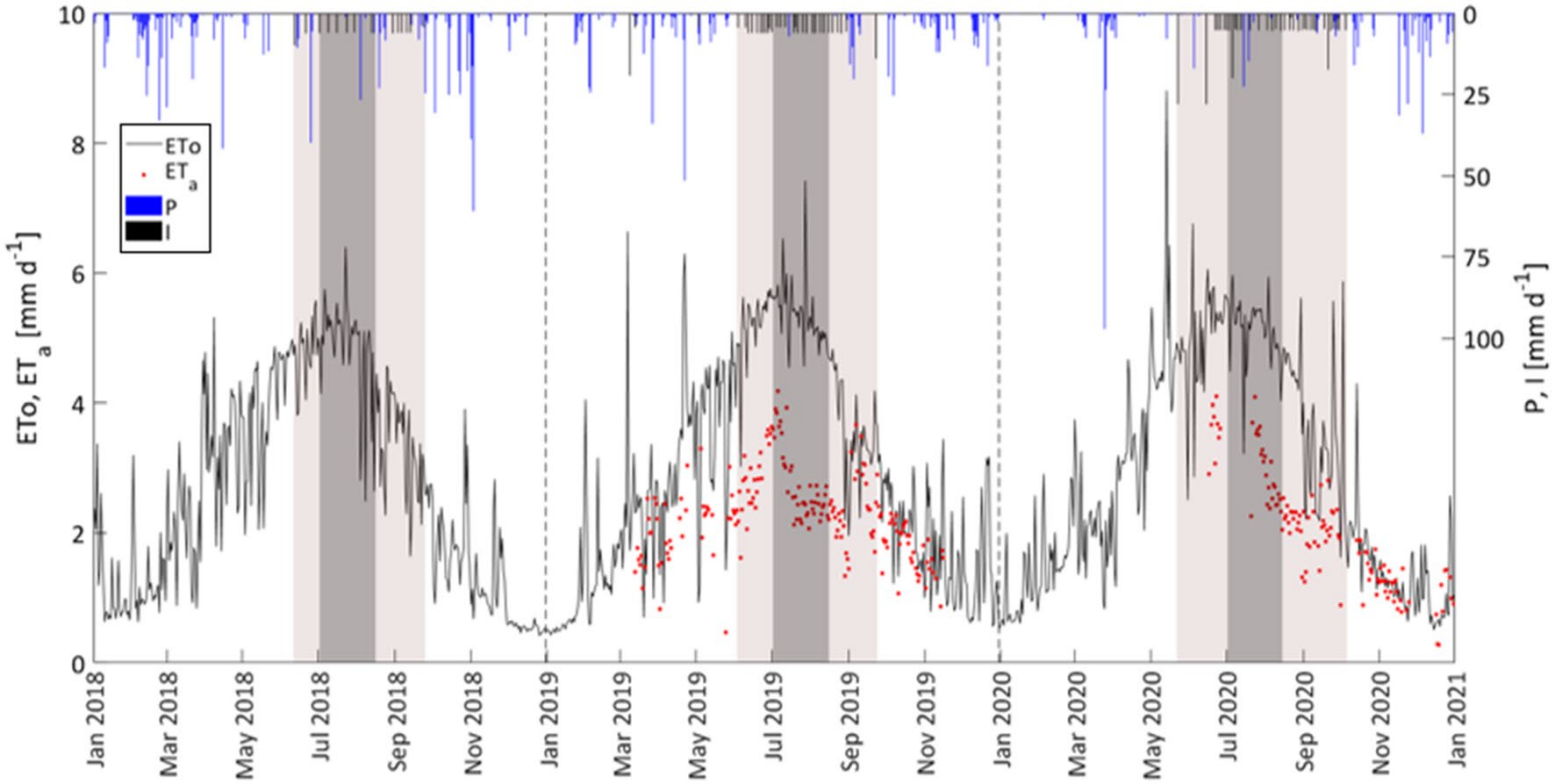
Temporal dynamic of crop reference evapotranspiration (continuous line), ETo, precipitation (blue bars), P, and amount of irrigation (black bars), I from 2018 to 2020. Available values of actual crop evapotranspiration (red dots), ETa, are also shown. The light box indicates the irrigation season, while the dark box identifies the period of application of water stress

**Fig. 3:**
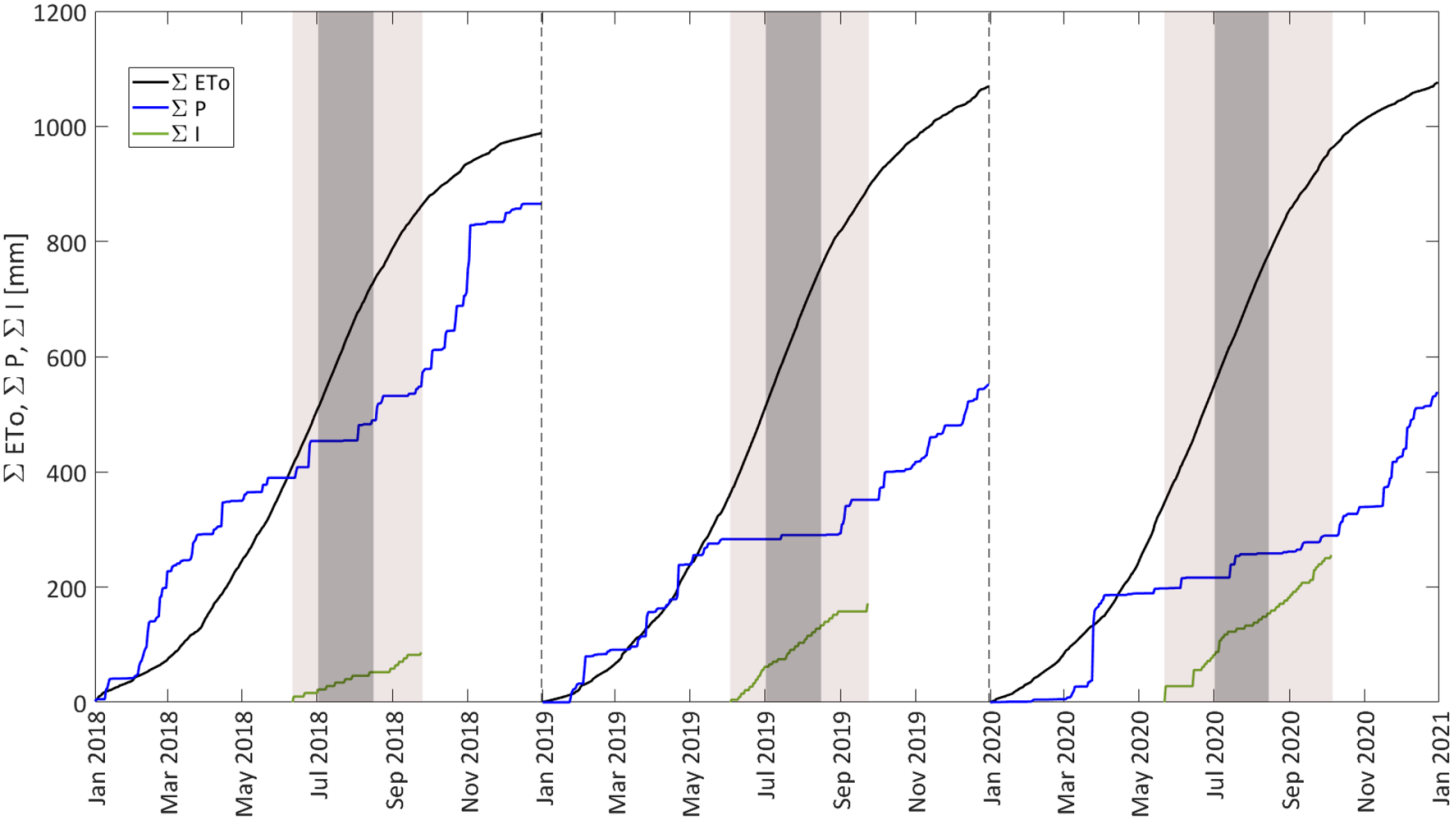
Cumulative precipitation, $\sum P$, crop reference evapotranspiration, $\sum {ETo}$, and irrigation, $\sum I$, distributions during 2018–2020. The light box indicates the irrigation season, while the dark box identifies the period of application of water stress

When considering the yearly cumulated precipitation, a total of 865 mm was recorded in 2018, and only 551 mm and 577 mm in 2019 and 2020. On the other hand, the yearly crop reference evapotranspiration resulted in 988 mm in 2018, 1069 mm in 2019 and 1076 mm in 2020 (Fig. 3), as a consequence of the relatively higher daily ETo values registered in 2019 and 2020.

For the three investigated years, Table 2 indicates the periods before, during and after irrigation season, for each of which summarizes the cumulated precipitation, ∑P, the amount of irrigation, ∑I, the crop reference evapotranspiration, ∑ETo, and the corresponding pluviometric deficits ∑(P-ETo). During the irrigation seasons, the lowest pluviometric deficit, equal to − 293 mm, was observed in 2018; on the other hand, irrigation seasons 2019 and 2020 were characterized by higher pluviometric deficit, with values equal to − 462 mm and − 523 mm, respectively, which suggested to anticipate the start of irrigation season and to increase the applied volumes.

**Table 2:** Cumulated annual precipitation, $\sum P$ amount of irrigation, $\sum I$, crop reference evapotranspiration, $\sum {{\text{ET}}_{0} }$, and pluviometric deficit, $\sum {\left( {P - ET_{0} } \right)}$, in the three years before, during and after irrigation seasons

Year	Date	Duration	Ʃ P	Ʃ I	Ʃ ET_0_	Ʃ (P-ET_0_)
		[d]	[mm]	[mm]	[mm]	[mm]
	01 Jan–11 Jun	162	390	–	411	– 21
2018	12 Jun–24 Sep	105	158	87	451	– 293
	25 Sep–31 Dec	98	317	–	127	190
	01 Jan–03 Jun	154	283	–	362	– 79
2019	04 Jun–23 Sep	112	68	172	530	– 462
	24 Sep–31 Dec	99	199	–	177	22
	01 Jan–22 May	143	198	–	347	– 149
2020	23 May–05 Oct	136	92	256	615	– 523
	06 Oct–31 Dec	87	248	–	115	133

**Fig. 4:**
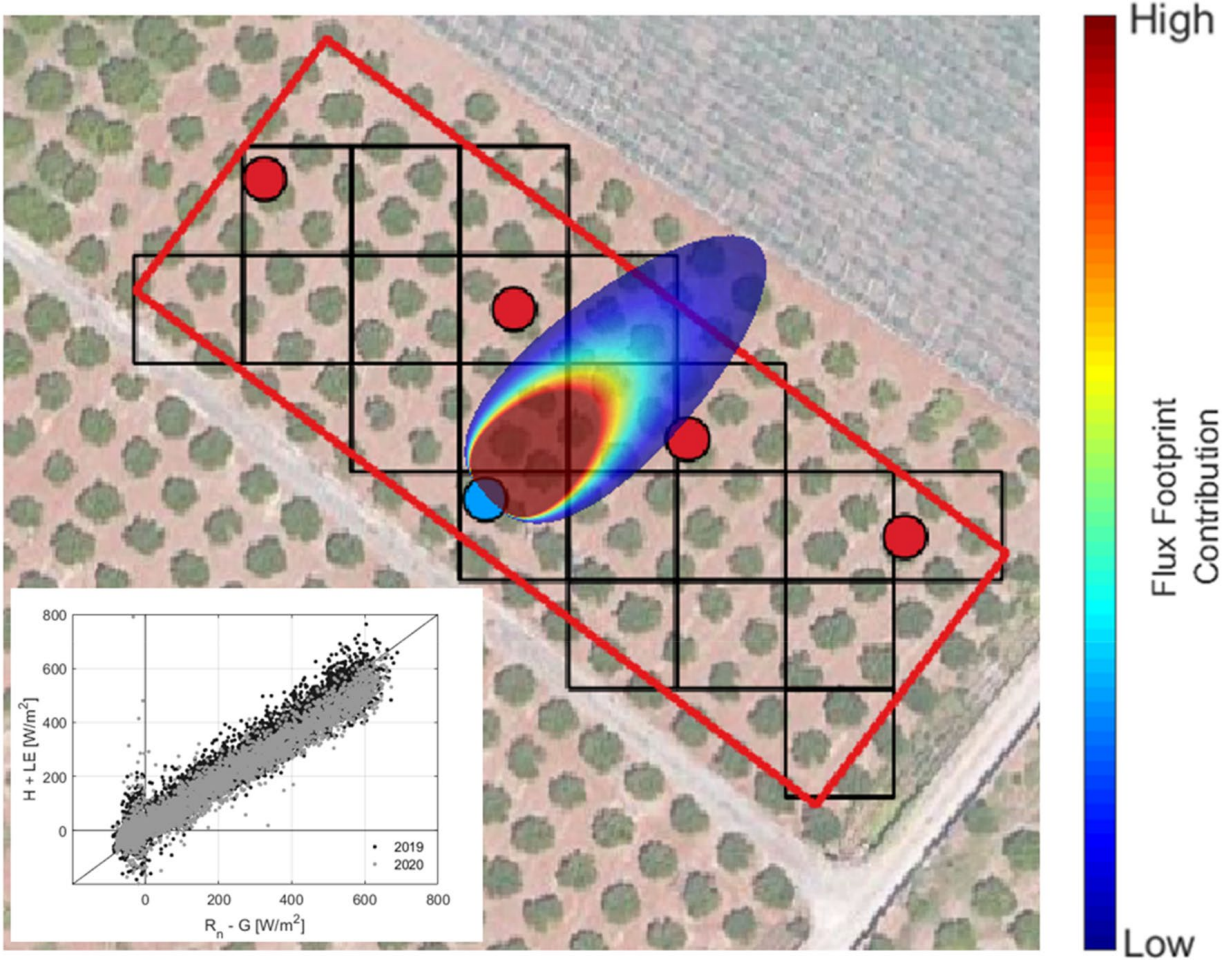
Map of the experimental plot showing the EC tower footprint. The inner box shows the scatterplot between hourly (H + LE) and (Rn – G) fluxes measured in 2019 (black dots) and 2020 (gray dots)

The footprint of the flux tower, which identifies the area on the ground encompassing at least 70% of the flux source, is shown in Fig. 4; the footprint was obtained based on the model proposed by Kljun et al. (2015) and considering the dominant wind speed of 1.7 m s^-1^ with a direction of 45° (NE). The accuracy of balance closure was verified based on the closure ratio, *CR,* (Prueger et al. 2005) computed only from the subset of hourly data with R_n_ ≥ 100 W m^−2^, whose values resulted equal to 0.98 in 2019 and 0.88 in 2020.

Daily actual evapotranspiration, ET_a_ measured with the EC tower only in 2019 and 2020 (Fig. 2), resulted in general lower than daily ETo, except during or immediately after rainy days, as a consequence of the higher contribution of evaporation component (French et al. 2020). EC system monitoring was interrupted from March to June 2020, due to the instrument failure occurring during the COVID-19 pandemic lockdown.

**Fig. 5:**
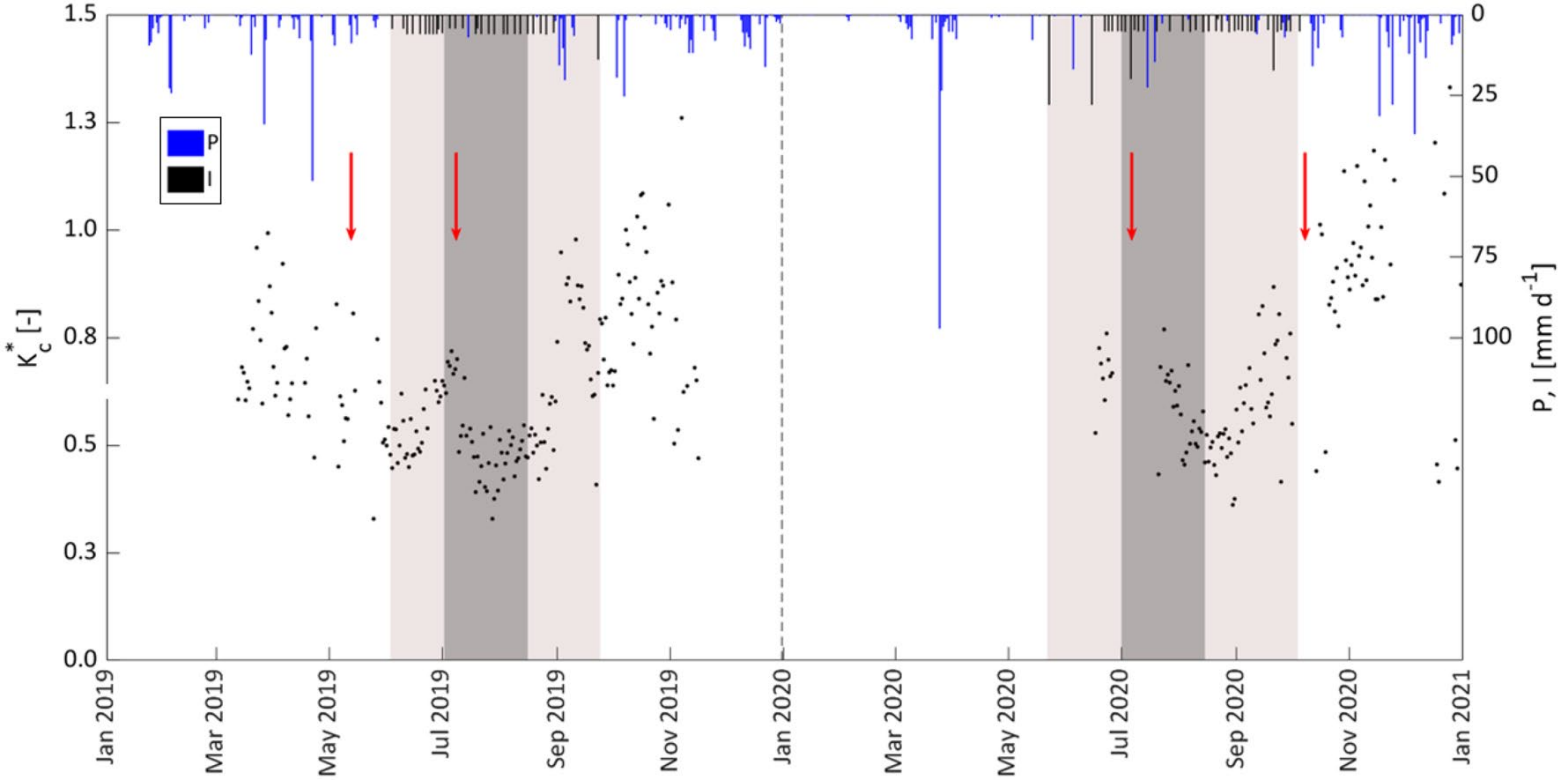
Values of the ratio between measured ET_a_ and ETo, ${K}_{c}$, in 2019 and 2020. The light box indicates the irrigation season, while the dark one identifies the period of water deficit application. Red arrows indicate the days in which weeds were cut down

The temporal dynamic of the ratio between ET_a_ and ETo, calculated after excluding the rainy days (P < 2.5 mm) from the dataset of measured ET_a_, is shown in Fig. 5. This ratio represents the actual crop coefficient,${\mathrm{K}}_{\mathrm{c}}^{*}$, represented by the product $, {\mathrm{K}}_{\mathrm{c}}^{*}$=K_c_*K_s_, between the standard unstressed crop coefficient, K_c_, and the water stress coefficient, K_s_. The latter is lower than the unit only in periods of crop water stress (generally occurring from the beginning of July to mid-August), and equal to the unit when soil water contents did not limit crop transpiration (absence of crop water stress). The values of ${\mathrm{K}}_{\mathrm{c}}^{*}$ were characterized by a quite high variability, with trends decreasing in spring and rising since the end of summer, up to values even higher than the unit. Moreover, the rapid decline of ${\mathrm{K}}_{\mathrm{c}}^{*}$ observed during the period of water deficit application (Fig. 5) was due to the contextual effect of the weeds removal from the soil surface (red arrows) and the limited water supply.

### Remote sensing data

**Fig. 6:**
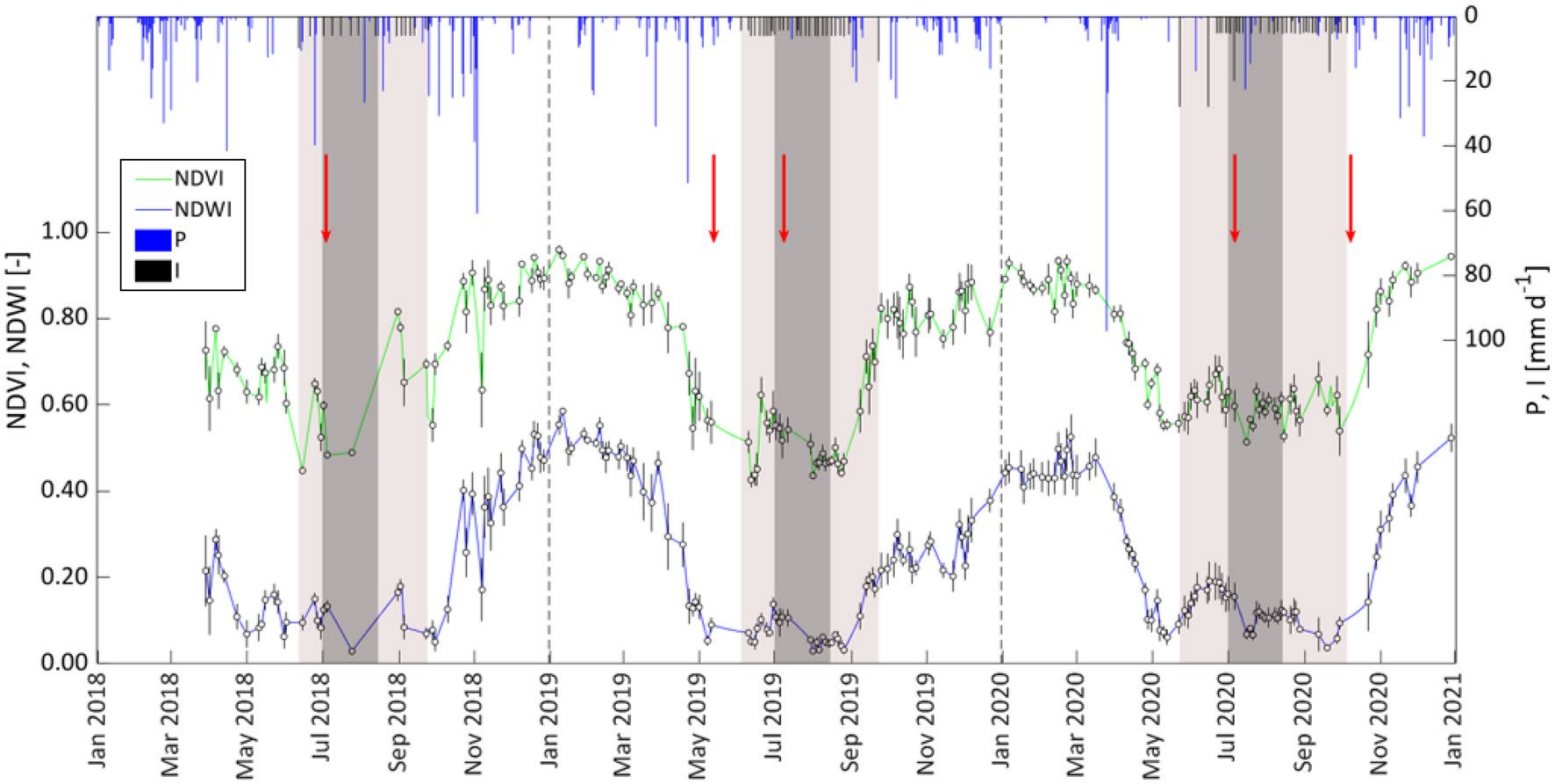
Temporal dynamic of average NDVI and NDWI, precipitation, P, and the amount of irrigation, I, for the investigated period (2018–2020). The light box indicates the irrigation season, while the dark box identifies the period of application of water stress. Red arrows indicate the days in which weeds were cut down

For the three investigated years, Fig. 6 shows the temporal dynamics of vegetation indices (NDVI and NDWI) retrieved from the Sentinel-2 clear-sky images database; the average and standard deviation of both VIs were determined by considering the four pixels containing the trees in which the soil water content sensors were installed. The generally low standard deviations characterizing both the VIs indicated that the four pixels were almost homogeneous. For both the VIs, the annual trends resulted quite similar, with values in winter generally higher than in summer. The occurrence of rainfall events during irrigation seasons 2018 and 2020 (Table 2) determined rising NDVI values, due to the rapid germination and emergence of spontaneous weeds on the soil surface. On the other hand, during irrigation seasons 2019, the lower amount of rainfall associated with the subsurface drip irrigation system did not drive the emergence of spontaneous weeds and determined, after weeding, the progressive decline of NDVI from 0.62 to 0.43. During the periods from late fall to early spring, NDVI resulted in slightly variable and assumed values up to 0.90, due to vigorous vegetation cover in the tree rows caused by the presence of uncut weeds. The values of NDWI, normally used to monitor the moisture conditions of vegetation canopies, ranged between 0.01 and 0.60, being the higher values associated with high vegetation water content and coverage of a large part of the field, and the lower associated with low vegetation water content and sparse coverage. The possibility to jointly use NDVI and NDWI was, therefore, supposed effective to improve the evaluation of the crop coefficient in sparse orchards characterized by the sporadic presence of cover weeds.

**Fig. 7:**
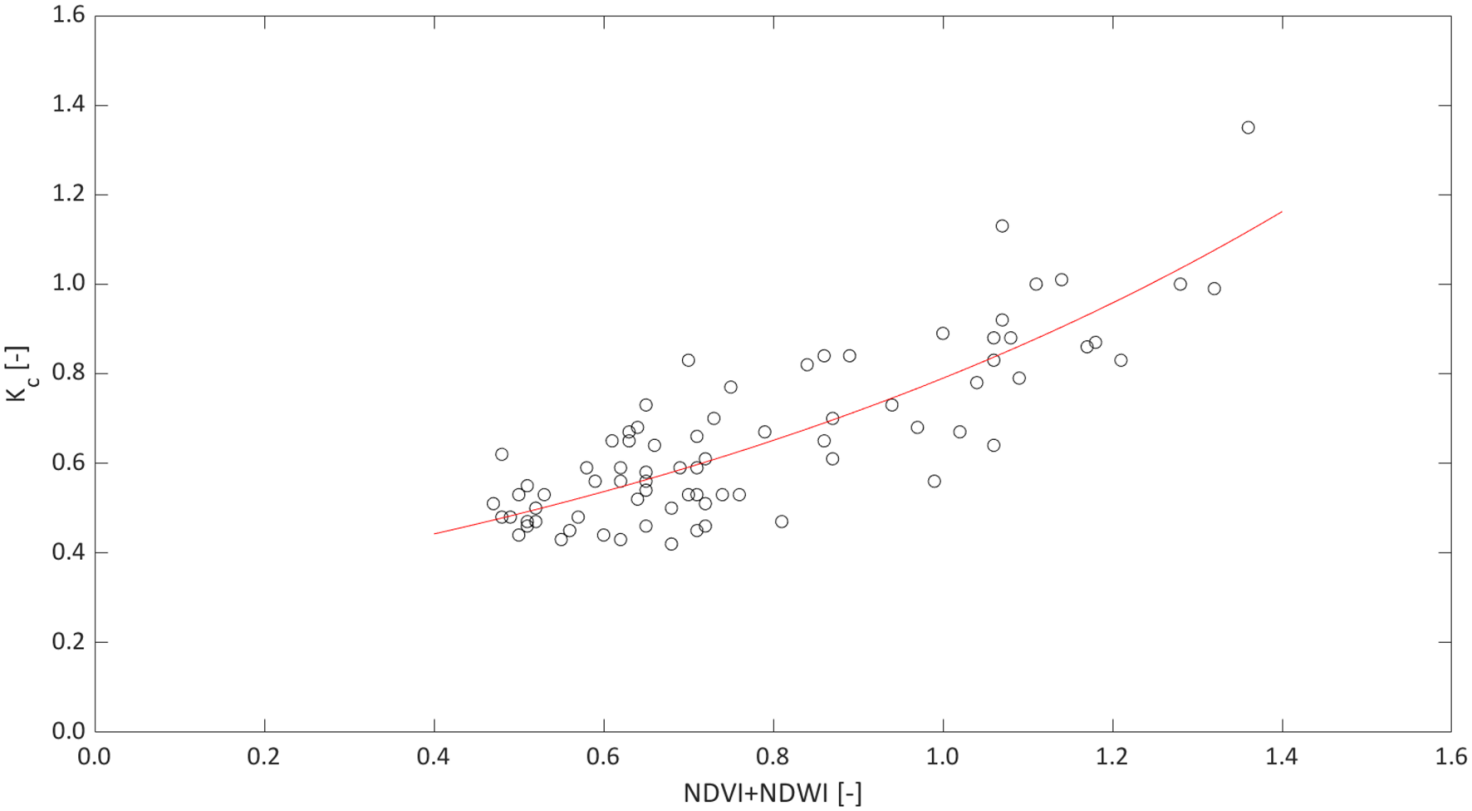
Predictive relationship to estimate the crop coefficient, *K*_*c*_, from the sum of NDVI + NDWI

To identify the relationship to predict the crop coefficient from the examined VIs, it was therefore assumed that the joint use of the examined indices (NDVI and NDWI) can better represent the actual field conditions characterized by sparse vegetation, the presence of transpiring weeds on the soil surface, and a limited period of water deficit application. Figure 7 shows the scatterplot representing the crop coefficient ${\mathrm{K}}_{\mathrm{c}}$, evaluated in the absence of crop water stress, as a function of the sum of NDVI and NDWI and the predictive relationship, expressed by the following exponential function characterized by R^2^ = 0.70:

12 $$K_{c} = a e^{{\left( {b\left( {NDVI + NDWI} \right)} \right)}} ,$$

in which *a* and *b* are two calibration coefficients equal to 0.304 and 0.939, respectively. To exclude the effects of the crop water stress in the predictive relationship, the periods of water deficit application (K_s_ < 1) were not included for this analysis.

**Fig. 8:**
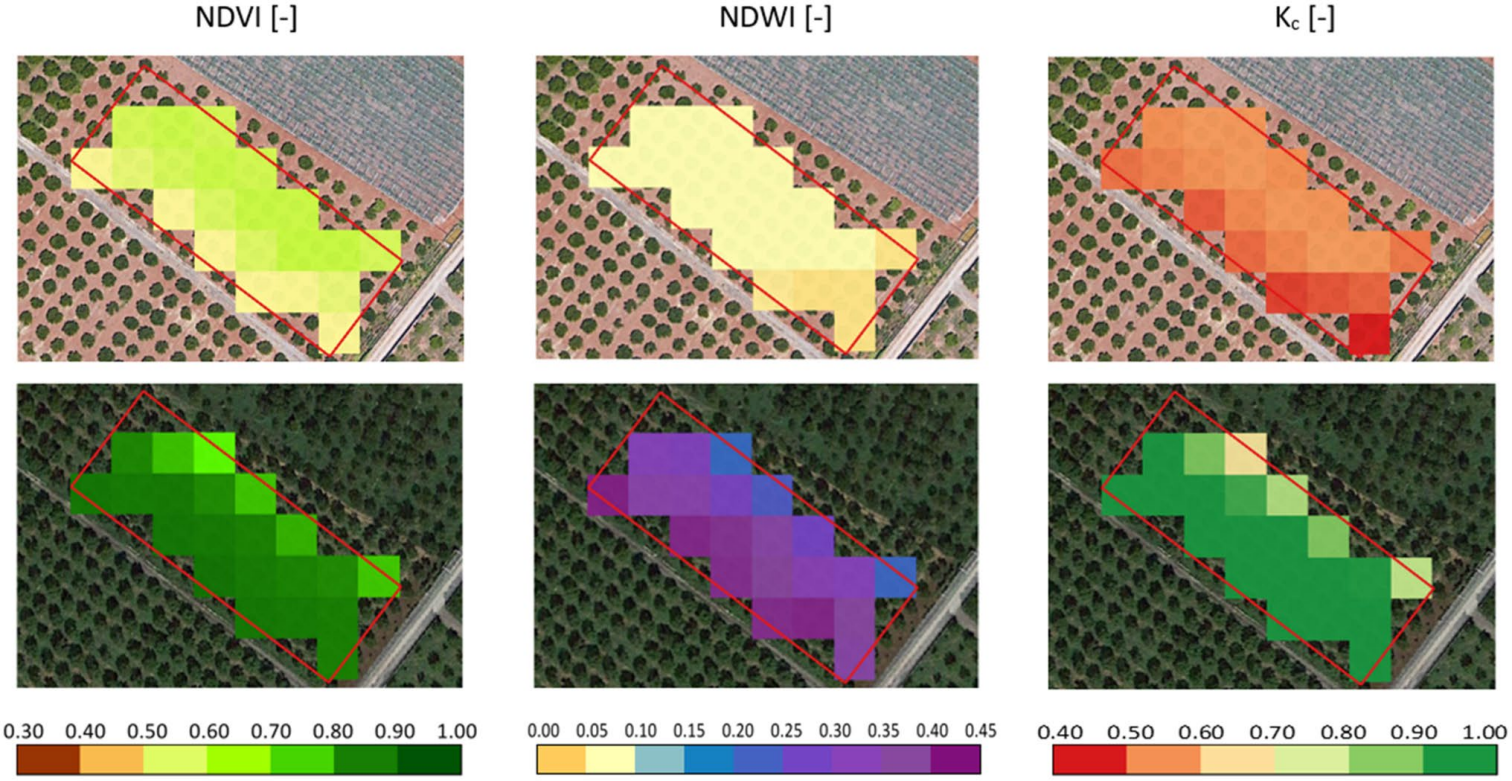
Maps of NDVI and NDWI obtained from Sentinel-2 acquisitions and corresponding *K*_*c*_ estimated from Eq. (12) in two days characterized by the absence (June 20, 2019, upper row) and the presence of transpirating cover weeds (December 4, 2019, lower row) on the soil surface

For the investigated field**, **Fig. 8 shows some examples of maps of NDVI and NDWI retrieved by Sentinel-2 acquisitions and the corresponding K_c_ estimated with Eq. 12 in two different days (June 20, 2019, and December 4, 2019) characterized by the absence (upper row) and the presence (lower row) of actively transpiring cover weeds on the soil surface. The sum of the two vegetation indices in the absence of cover weeds (NDVI_av_ = 0.59, NDWI_av_ = 0.10) resulted lower than that obtained under the presence of active cover weeds (NDVI__mean_ = 0.90, NDWI__mean_ = 0.36). The observed difference is due to the diverse spectral responses caused by the presence of weeds covering the soil among the tree rows and consequently, the average K_c_ estimated in the absence of cover weeds (K_c_ = 0.58) resulted lower than the corresponding obtained in the presence of actively transpiring weeds (K_c_ ~ 1.00).

**Fig. 9:**
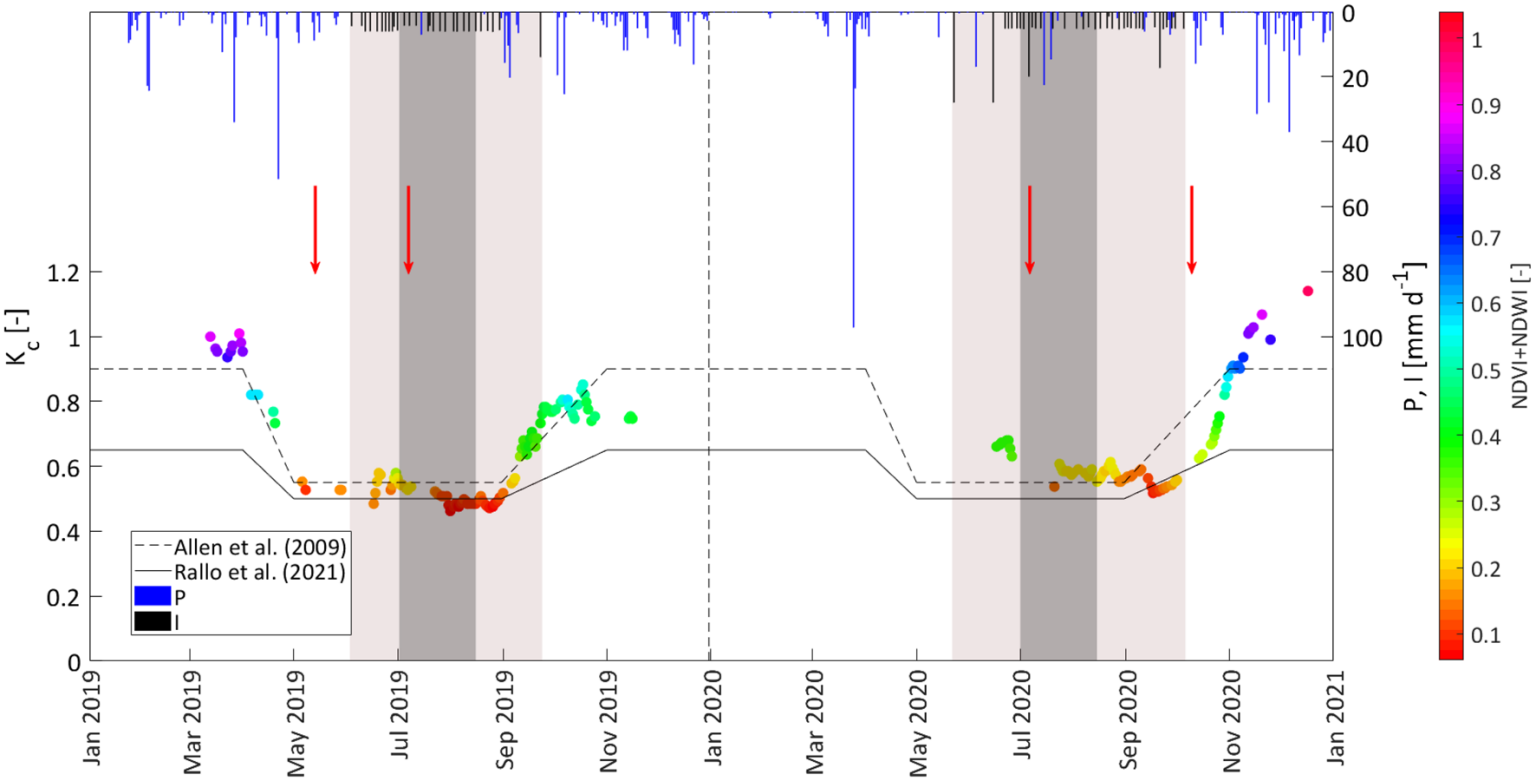
Comparison between crop coefficient estimated with Eq. (12) and the corresponding curves suggested by Allen et al. (2009) (dashed line) and Rallo et al. (2021) (continuous line). The colors associated with the experimental values depend on the combination of NDVI and NDWI. The light gray box indicates the irrigation season (IS), while the dark gray identifies the period of application of water deficit (DI). Precipitation, P (blue bars), and Irrigation events (black bars), I, are indicated in the secondary axes

In Fig. 9, the temporal trends of crop coefficient estimated with Eq. (12) were compared with those obtained from the literature for citrus orchards characterized by the presence (Allen and Pereira 2009) and absence (Rallo et al. 2021) of ground weeds. The colors associated with the estimated K_c_ values depend on the sum of NDVI and NDWI. As it can be observed, relatively higher K_c_ associated with the greater vegetation indices combination, with values, close to 1.0, were obtained from late fall to early spring, after the beginning of sprouting, while in the following stage, estimated K_c_ values were associated with the decreasing vegetation indices combination. On the other hand, during the irrigation season, under absent or scarce precipitation, the estimated K_c_, associated with the relatively lower combination of vegetation indices, resulted in about 0.55; finally, after the end of irrigation, the rising estimated K_c_, associated with increased values of the vegetation indices, can be justified by the emergency and development of ground weeds among the plant rows. In 2020, the late growth season started later than in 2019, as a consequence of the quite high pluviometric deficit characterizing the mid-season 2020.

### FAO-56 soil water balance model

For the examined field, the FAO-56 procedure was implemented in a spatially distributed mode to estimate the soil water balance with a 10 m spatial resolution. However, considering that SWC measurements were acquired in only four trees, while actual evapotranspiration measurements involved the entire field, the validation of the spatially distributed FAO-56 model was performed at field scale using the average of the output pixel values falling inside the perimeter of the study area. In this way, the results of the pixel-based FAO-56 procedure were aggregated at a field scale where evapotranspiration and SWC measurements were carried out.

**Fig. 10:**
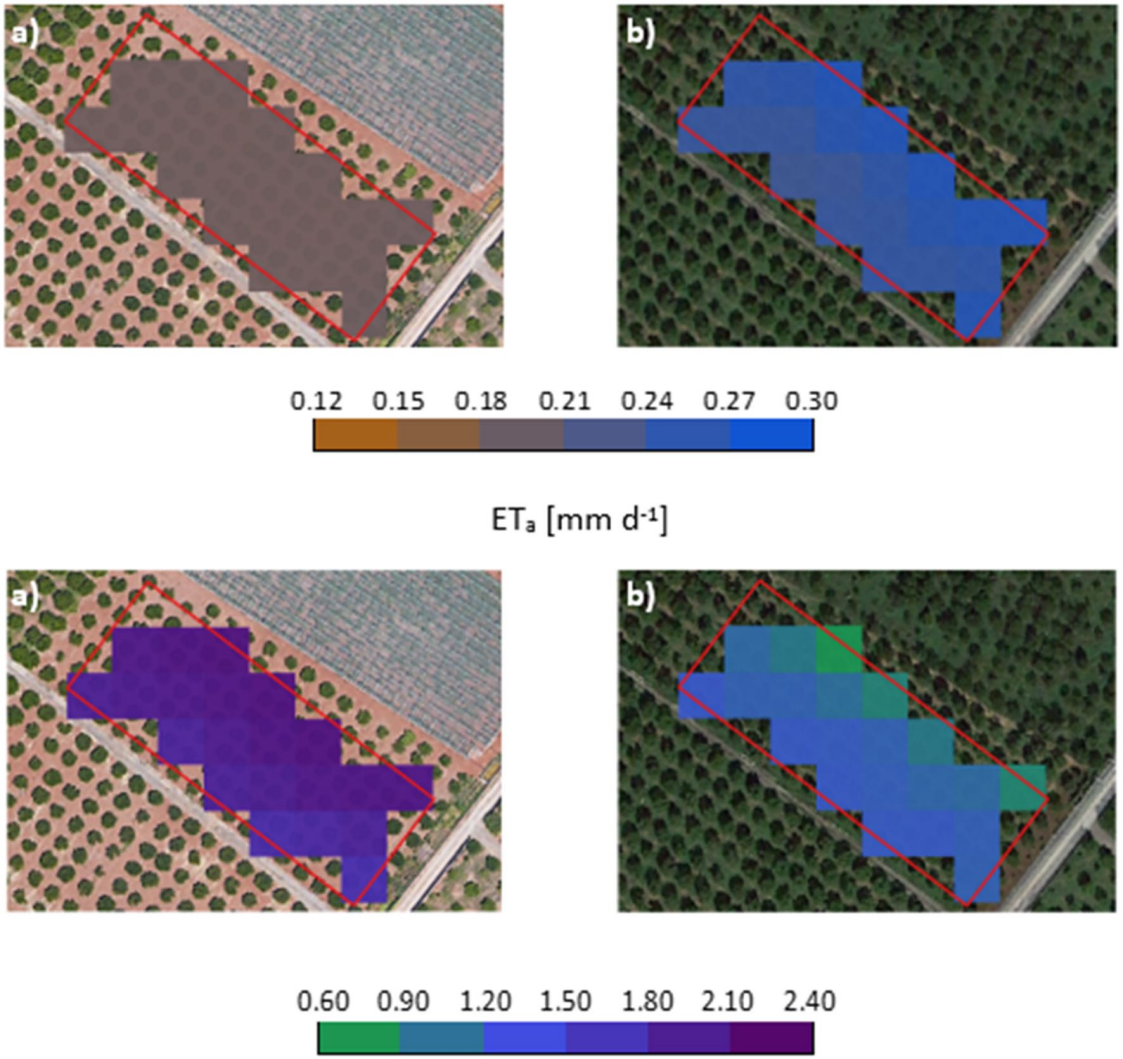
Examples of maps of simulated soil water content, SWC_sim_, and actual evapotranspiration, ET_a_, obtained in the absence (**a** June 20, 2019) and presence (**b** December 4, 2019) of active weeds among the tree rows

To give an example, Fig. 10 shows the maps of simulated soil water content (SWC_sim_) and actual evapotranspiration (ET_a_) retrieved from the application of the FAO-56 model on two different days, i.e., on June 20, 2019, and December 4, 2019, respectively, in the absence and presence of active weeds on the soil surface. During these two days, the average ET_a_ resulted equal to 3.27 mm d^−1^ and 1.27 mm d^−1^, with a standard deviation of 0.17 mm d^−1^ and 0.13 mm d^−1^, respectively. Moreover, on the same days, the average SWC_sim_ resulted equal to 0.19 ± 0.00 cm^3^ cm^−3^ and 0.27 ± 0.01 cm^3^ cm^−3^, respectively. The value of K_c_ was close to 1.0 on days in which weeds were present on the soil surface determining values of ET_a_ fairly close to the corresponding ETo.

**Fig. 11:**
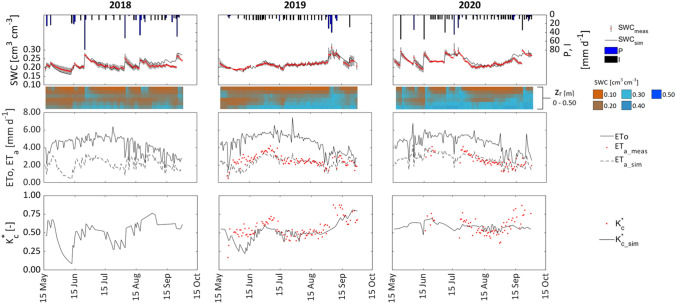
Temporal dynamics of average and standard deviation of measured SWC_meas_ and corresponding simulated, SWC_sim_, precipitation, P, amount of irrigation, I (upper row) and detail of soil water contents measured each 10 cm depth in the layer 0–50 cm (second row); temporal dynamics of crop reference evapotranspiration, ETo, and of measured, ET_a_meas_, and simulated actual evapotranspiration, ET_a_sim_, (third row); temporal dynamics of measured and estimated *K*_c_* (lower row) for the simulated periods of 2018, 2019 and 2020

The results of the FAO-56 model simulations applied at the field scale for the three years (from 17 May to 30 September) are shown in Fig. 11. The upper row illustrates the comparison between measured (SWC_meas_) and simulated (SWC_sim_) soil water contents, while the second row shows the soil water contents distribution in the soil layer 0–0.50 m where the active root system is developed. The daily values of measured SWC of the entire experimental field were calculated as the mean of the values acquired in layer 0–0.50 m by the four probes installed in the plot. A fairly good agreement can be observed between simulated and measured SWC in the root zone, even if local but negligible discrepancies can be noticed mainly after rainfall events and, for years 2018 and 2020, around the final periods of simulations, when a slight overestimation of simulated SWC occurred. When observing the temporal dynamics of SWC profiles, it is interesting to notice that water applications with the subsurface drip irrigation system increased only the SWC at depths ranging from 0.30 m to 0.50 m, whereas the upper soil layer remained generally dry. The third row of Fig. 11 shows the dynamic of crop reference evapotranspiration (ETo) in the three years, as well as measured (ET_a_meas_) and simulated (ET_a_sim_) actual evapotranspiration. For the two years in which measured ET_a_ values were available, the trends of simulated values followed, in general, those of the corresponding measured, even if a slight underestimation can be observed in the initial period of simulation of 2019, probably due to rapid depletion of soil water content consequent to the absence of rainfall events in the period. Finally, the lower row of Fig. 11 shows the trend of measured and simulated ${\mathrm{K}}_{\mathrm{c}}^{*}$, whose values resulted generally in a fairly good agreement. The predictive relationship, therefore, can represent a useful alternative to estimate the actual crop coefficient under the examined field conditions and the presence of temporarily active weeds among the plant rows.

The performance of the K_c_(VI) relationship implemented in the FAO-56 model was evaluated based on the statistical parameters associated with simulated and measured soil water contents and actual evapotranspiration, reported in Table 3 for the periods of observation. The model allows predictions of daily SWC very accurate, with RMSE equal to 0.01 cm^3^ cm^−3^ and the absence of bias, as a consequence of the substantial agreement between the average soil water contents measured in the root zone and the corresponding simulated by the model. The goodness of the proposed K_c_(VI) relationship is also confirmed by the quite high R^2^ values, the b coefficient marginally higher than the unit and the always positive NSE. However, the negative values of PBIAS obtained in the three years of simulation indicate a weak tendency of simulated values to be higher than the corresponding observed. On the other hand, the simulated values of actual evapotranspiration, ET_a_, were characterized by RMSE equal to 0.57 and 0.40, MBE of -0.26 mm d^−1^ and -0.01 mm d^−1^ and PBIAS of 10.4 and 0.2 for 2019 and 2020, respectively. Despite these values indicating that mainly in 2019 the simulated actual evapotranspiration resulted slightly underestimated, the high values of the regression coefficient b (higher than 0.88), and the positive NSE index, allow considering acceptable, for practical application aimed at irrigation scheduling, the performance of the model to estimate ET_a_ when assuming, as an input variable, the crop coefficients retrieved by Sentinel-2 images.

**Table 3:** Results of the statistical analysis to check the FAO-56 model performance

	SWC					
	*RMSE*	*MBE*	*PBIAS*	*R*^*2*^	*b*	*NSE*
	[cm^3^/cm^3^]	[cm^3^/cm^3^]	[%]	[–]	[–]	[–]
2018	0.01	0.00	− 1.30	0.74	1.01	0.40
2019	0.01	0.00	− 0.49	0.86	1.00	0.85
2020	0.01	0.00	− 2.11	0.80	1.02	0.76

	ET_a_					
	[mm/day]	[mm/day]	[%]	[−]	[–]	[–]
2019	0.57	− 0.26	10.36	0.44	0.88	0.21
2020	0.40	− 0.01	0.22	0.70	0.98	0.68

## Discussion and conclusions

To increase irrigation water use efficiency of woody crops, it is necessary to improve the irrigation infrastructure and method, as well as to adopt site-specific strategies of water management to control crop water status and limit the water supply to the amounts requested by the plants. To this aim, a multidisciplinary approach is necessary to exploit the most advanced irrigation techniques and crop monitoring with recent earth observation technologies and agro-hydrological modeling.

In the present study, the simultaneous availability of high-resolution satellite images and site-specific observations acquired with eddy covariance and weather station systems allowed simulating with high precision the water and energy exchanges of a citrus orchard equipped with a subsurface drip irrigation system.

For the study area, the use of a subsurface drip irrigation system, with laterals buried 30 cm below the soil surface, ensured the efficiency either in terms of conveyance and distribution of irrigation water (Nair et al. 2013; Martinez-Gimeno et al. 2018); during irrigation seasons, the generally dry topsoil limited soil evaporation and the presence of actively transpiring spontaneous vegetation. On the other hand, using the traditional surface drip irrigation systems would have determined the increase of soil evaporation and the growth of transpiring weeds around the wetted zone and, consequently, the rise of K_c_. However, during irrigation seasons 2018 and 2020, the occurrence of rainfall events determined the germination of spontaneous vegetation, which contributed to increasing the orchard evapotranspiration fluxes. Under these conditions, the use of tabular crop coefficient could have led to an inaccurate estimation of actual crop evapotranspiration, while the estimation of site-specific crop coefficient contributed to enhancing the results of modeling applications.

The availability of measurements acquired by the eddy covariance tower and the weather station in 2019 and 2020, provided the source of data to estimate the crop coefficient as the ratio between ground-based ET_a_ retrieved in the absence of water stress and ETo (Calera et al. 2017). The occurrence of crop water stress was verified based on the measurements of midday stem water potential which allowed identifying the critical threshold of soil water content, equal to 0.21 cm^3^ cm^−3^, below which, relative crop transpiration starts to decrease (Franco et al. 2021; 2022). This value resulted quite similar to that identified by Puig-Sirera et al. (2021b) for a citrus orchard (*Citrus Clementina*, Hort ex Tan.) in Spain. Even though crop water deficit was scheduled from the beginning of July to mid-August (phase II of the crop growth stage), two heavy rainfall events registered in mid-July 2020 delayed the occurrence of soil water deficit, limiting the periods characterized by K_s_ < 1. During irrigation seasons, excluding the short periods of crop water stress, the values of crop coefficient ranged between 0.47 and 0.76, in line with the tabulated values of 0.50 and 0.82 suggested by Allen et al. (1998) for a citrus orchard characterized by a fraction cover of 30%, respectively, in absence and presence of active ground cover or weeds. Moreover, an indicative value of the mid-season crop coefficient, equal to 0.55 ± 0.5, has been recently confirmed by Rallo et al. (2021) for low-density citrus orchards characterized by a fraction cover between 25 and 40% and with a plant height between 2.3 and 4.5 m.

The values of crop coefficient were obtained by the combination of two vegetation indices, NDVI and NDWI, the first of which is representative of the plant vigor and vegetative fraction; relatively higher tree canopy coverage increases the plant transpiration component, while lower values determine its decrease. Higher basal crop coefficient, K_cb_, associated with the fraction cover is consistent with the methodology proposed by Allen and Peeira (2009) and Pereira et al. (2020) allowing the evaluation of K_cb_ from the fraction of ground cover and plant height. On the other hand, irrigation frequency should mainly affect the temporal dynamic of the stress coefficient K_s_ and not the values of K_cb_.

The dispersion of the measured crop coefficient tended to increase in periods characterized by frequent rainfall events as a consequence of the rapid growth of active weeds on the soil surface (Fig. 5). On the other hand, the removal of the weeds determined the rapid reduction of the associated transpiration and the decline of K_c_.

The use of remote sensing (RS) technologies for a range of agricultural applications have been exponentially increased over the past few decades (Khanal et al. 2020), and the possible applications will surely furtherly grow in the coming years. The challenges associated with the use of RS for estimating and mapping single (K_c_) and basal (K_cb_) crop coefficients based on vegetation indices have been recently discussed by Pôças et al. (2020), who concluded that the operative advantages of such an approach are not questioned and the technology is mature to support irrigation scheduling. In this direction, the present study demonstrated the suitability of a combination of VIs retrieved by Sentinel-2 multispectral images to indirectly estimate the spatio-temporal variability of the crop coefficient (K_c_) in a citrus orchard, during the different stages of crop growth. For the three examined years, the available satellite clear-sky scenes resulted, on average, equal to 60–70 per year, with an average revisiting time, during the irrigation season of 4 days, thus ensuring an almost continuous tracking of the orchard temporal variability. Moreover, in the view to promote precision irrigation, the quite high resolution of Sentinel-2 scenes (10–20 m) can allow detecting the possible intra-field spatial variability, which can be used to divide the farm into homogeneous zones requiring differentiated management.

Even if the literature reports a large number of empirical K_cb_–VI relationships, developed for different crops and under different operating conditions, the proposed non-linear relationship (Eq. 12) included a combination of two indices, namely the sum between NDVI and NDWI, the first of which to account for the plant vigor, LAI and fraction cover, while the second, depending on the shortwave reflectance, is sensitive to the change of moisture conditions of the vegetation canopy (Gao 1996), including the effects of spontaneous vegetation covering the soil surface.

Values of estimated K_c_ close to 1.0 were in general obtained from late fall to early spring, under the greater vegetation indices combination (Fig. 9), whereas they resulted to vary around 0.55 during the mid-seasons in which the combination of vegetation indices was relatively lower; finally, after mid-season, the values of K_c_ associated to the increasing values of the vegetation indices combination tended to rise gradually, following the emergency and development of ground weeds among the plant rows. In other words, the K_c_ values for the mid-season, when soil evaporation and weed transpiration were limited, resulted lower than the segments relative to the late and the non-growing seasons, in which rainfall and evapotranspiration from the soil surface occur. Similar annual patterns of crop coefficient have been recently reported by Puig-Sirera et al. (2021a) for an olive orchard and by Segovia-Cardozo et al. (2021) in a citrus orchard in which, focusing on the full year and not only on the growing season, it was observed that K_c_ values in non-growing periods resulted in higher values than in the growing period, due to the large contribution of soil evaporation.

The VIs-based K_c_, implemented in the spatially distributed FAO-56 model under the real irrigation strategy, allowed the precise estimations of daily and seasonal soil water status and actual crop evapotranspiration (Calera et al. 2017; Rallo et al. 2017), also accounting for the presence of temporarily transpiring weeds, which increased the velocity with which water was depleted from the root zone, with the consequent reduction of crop water availability and the need of more frequent watering. Considering that simulated soil water contents during the examined periods exceeded the soil field capacity (*SWC*_*fc*_ = 0.28 cm^3^ cm^−3^) quite rarely and only after a few abundant rainfall events (Fig. 11), deep percolation was neglected. The fairly good agreement between estimated and measured soil water content and actual crop evapotranspiration at plot level (Table 3) confirmed the suitability of the proposed K_c_(VI) relationship to characterize the biophysical characteristics of the land surface and to improve the estimation of actual evapotranspiration.

Even if the crop coefficients tabulated for the different stages of citrus crop growth, on average, were confirmed in the present research, the possibility of using site-specific crop coefficients, accounting for local and time-variable conditions occurring in the field can contribute to improving the crop water requirement predictions and irrigation scheduling. Moreover, the proposed K_c_(VI) relationships, implemented in a GIS environment, can also contribute to driving the implementation of precision irrigation strategies accounting for the actual field spatial variability.

## Data Availability

The data supporting the findings of this study are available upon request from the author.
